# Supplementary material for: Conformational Remodeling and Allosteric Regulation Underlying EGFR Mutant-Induced Activation: A Multi-Scale Analysis Using MD, MSMs, and NRI
Source: Int J Mol Sci. 2025 Jun 27;26(13):6226. doi: 10.3390/ijms26136226 (PMC12250381; doi:10.3390/ijms26136226)
Supplement: Supplementary file 1 [file ijms-26-06226-s001.zip › ijms-3700323-supplementary.pdf]

# Conformational Remodeling and Allosteric Regulation Underlying EGFR Mutant-Induced Activation: A Multi-Scale Analysis Using MD, MSMs, and NRI

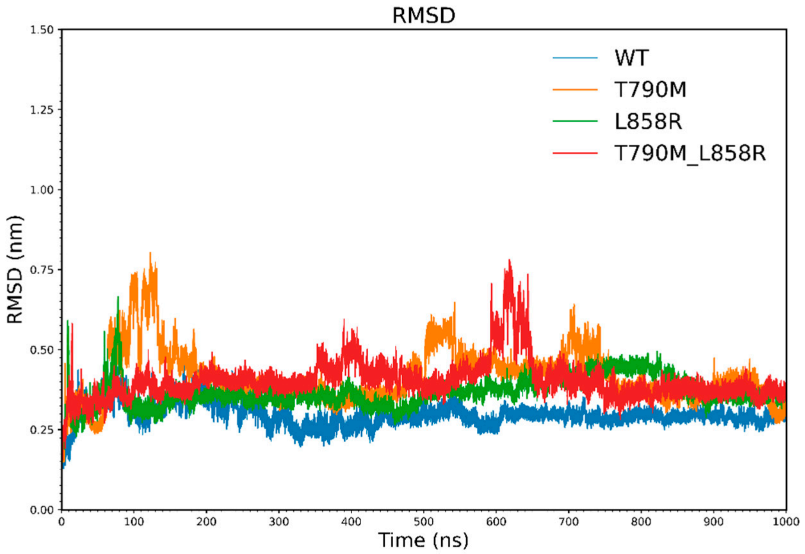


Figure S1. RMSD values of backbone atoms of WT-EGFR, T790M-EGFR, L858R-EGFR, and T790M+L858R-EG FR as a function of time with respect to the initial structure.

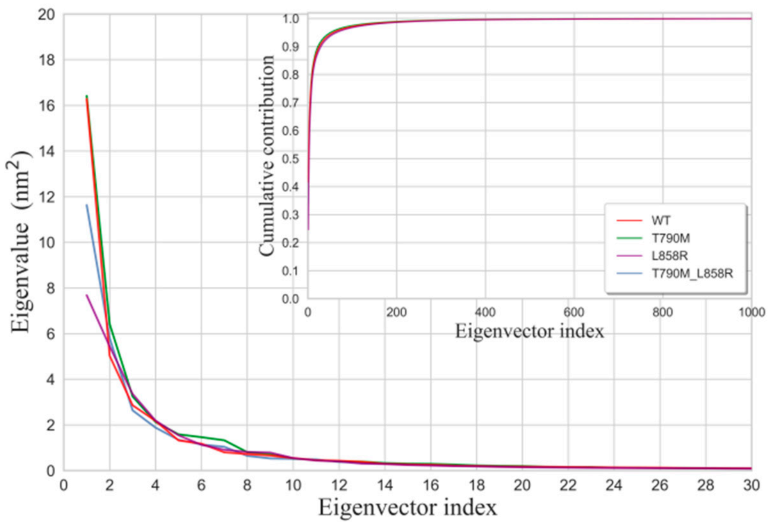


Figure S2. Eigenvalues of the first 30 eigenvectors of WT-EGFR, T790M-EGFR, L858R-EGFR, and T790M_ L858R-EGFR (main plot) and cumulative contribution to TMSF for all eigenvectors (inset) by python scripts.

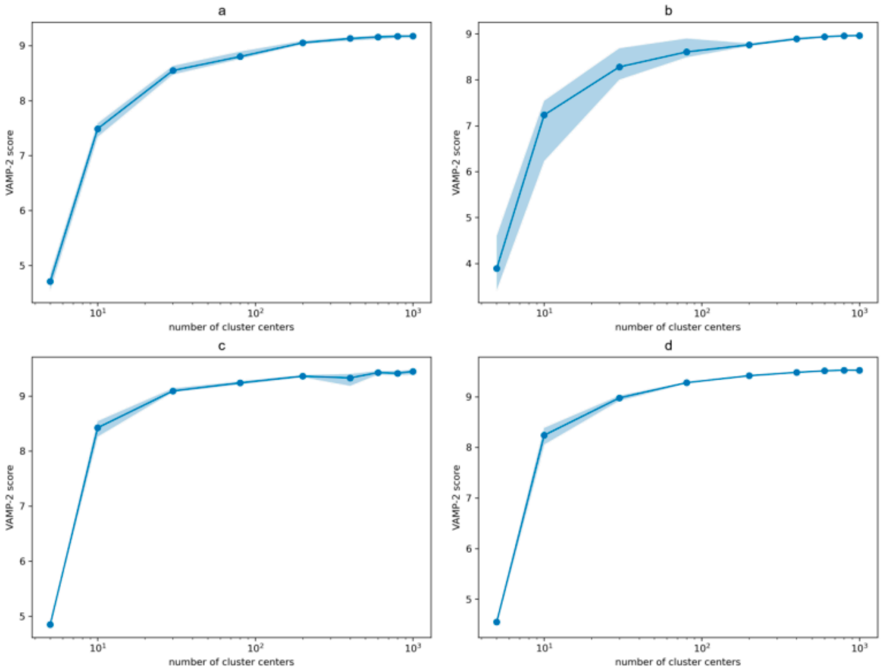


Figure S3. VAMP-2 scores for different numbers of clustered centres. (a) WT-EGFR (b) T790M-EGFR (c) L858R -EGFR (d) T790M_L858R-EGFR.

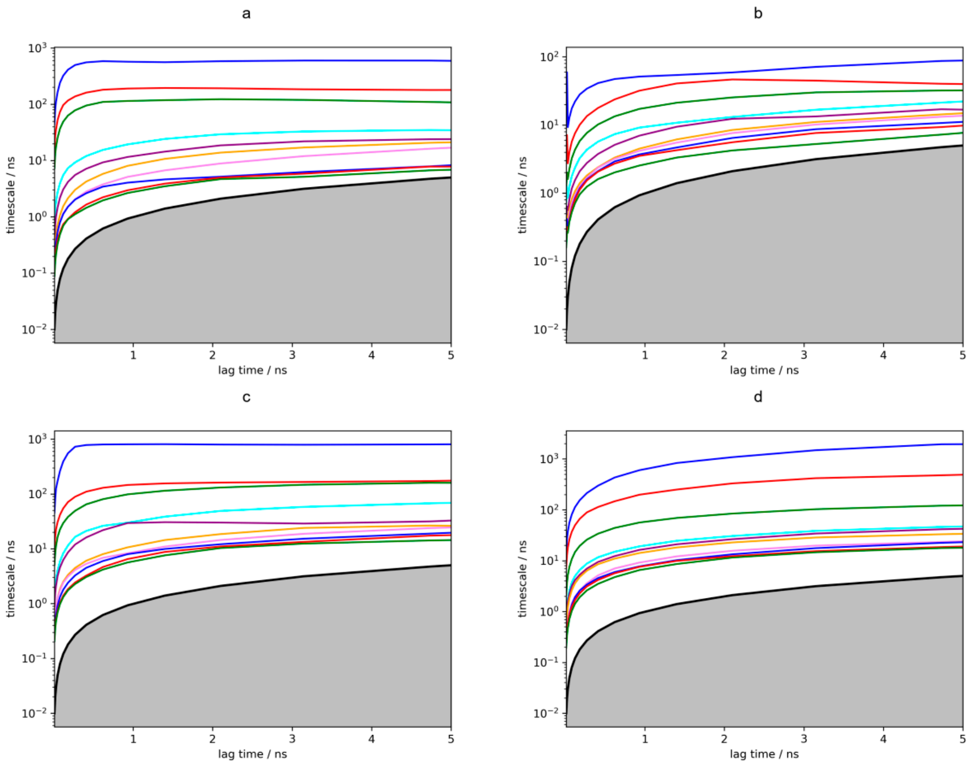


Figure S4. Implied time scales. (a) WT-EGFR (b) T790M-EGFR (c) L858R-EGFR (d) T790M_L858R-EGFR.
